# K^+^ channelepsy: progress in the neurobiology of potassium channels and epilepsy

**DOI:** 10.3389/fncel.2013.00134

**Published:** 2013-09-13

**Authors:** Maria Cristina D'Adamo, Luigi Catacuzzeno, Giuseppe Di Giovanni, Fabio Franciolini, Mauro Pessia

**Affiliations:** ^1^Faculty of Medicine, Section of Human Physiology, Department of Internal Medicine, University of PerugiaPerugia, Italy; ^2^Istituto Euro Mediterraneo di Scienza e Tecnologia, IEMESTPalermo, Italy; ^3^Dipartimento di Biologia Cellulare e Ambientale, Università di PerugiaPerugia, Italy; ^4^Department of Physiology and Biochemistry, University of MaltaMsida, Malta

**Keywords:** Potassium channels: [Kv1, Kv2, Kv3, Kv4, Kv8, Kv11(HERG), K_Ca1.1_, Kvβ1, Kvβ2, KChIP LGI1, Kir1-Kir7 (GIRK, K_ATP_)], temporal lobe epilepsy, autism–epilepsy, channelopathies

## Abstract

K^+^ channels are important determinants of seizure susceptibility. These membrane proteins, encoded by more than 70 genes, make the largest group of ion channels that fine-tune the electrical activity of neuronal and non-neuronal cells in the brain. Their ubiquity and extremely high genetic and functional diversity, unmatched by any other ion channel type, place K^+^ channels as primary targets of genetic variations or perturbations in K^+^-dependent homeostasis, even in the absence of a primary channel defect. It is therefore not surprising that numerous inherited or acquired K^+^ channels dysfunctions have been associated with several neurologic syndromes, including epilepsy, which often generate confusion in the classification of the associated diseases. Therefore, we propose to name the K^+^ channels defects underlying distinct epilepsies as “*K^+^ channelepsies*,” and introduce a new nomenclature (e.g., K*x.y*-*channelepsy*), following the widely used K^+^ channel classification, which could be also adopted to easily identify other *channelopathies* involving Na^+^ (e.g., Na_v_*x.y*-*phenotype*), Ca^2+^ (e.g., Ca_v_*x.y*-*phenotype*), and Cl^−^ channels. Furthermore, we discuss novel genetic defects in K^+^ channels and associated proteins that underlie distinct epileptic phenotypes in humans, and analyze critically the recent progress in the neurobiology of this disease that has also been provided by investigations on valuable animal models of epilepsy. The abundant and varied lines of evidence discussed here strongly foster assessments for variations in genes encoding for K^+^ channels and associated proteins in patients with idiopathic epilepsy, provide new avenues for future investigations, and highlight these proteins as critical pharmacological targets.

## Introduction

Epilepsy is a brain disorder due to abnormal firing of neuronal networks in the brain that often causes convulsions, muscle spasms, and loss of consciousness. Seizures sometimes cause brain damage, particularly if they are severe. More than 2 million people in the United States—*about 1 percent*—have experienced an unprovoked seizure or been diagnosed with epilepsy. For about 80 percent of those diagnosed with epilepsy, seizures can be controlled pharmacologically, or treated surgically. However, the rest of people with epilepsy—*intractable epilepsy*—will continue to experience seizures even with the best available treatment. Although the causes of epilepsy are numerous, the fundamental disorder is secondary to abnormal synchronous discharges of a network of neurons, either due to neuronal membrane instability, or an imbalance between excitatory and inhibitory influences. Neurons store and convey information in the form of electrical impulses generated by ion channels. Neuronal excitability can be controlled by both the intrinsic activity of K^+^ channels and the receptors-mediated modulation of their activity (Pessia et al., 1994; Imbrici et al., 2000; Pessia, 2004; D'Adamo et al., 2013). Their opening and resulting outward K^+^ flux dampen neuronal excitability and therefore they are viewed as inhibitory channels. However, contrary to this general notion, increased K^+^ channel activity may also result in enhanced cell excitability. Hence, *K^+^ channels are critical for neuronal excitability*, as they control the resting membrane potentials and enable rapid repolarization of the action potentials. Moreover, they are essential effectors of neurotransmitter-mediated signaling, regulator of Ca^2+^ homeostasis and cell survival. To fulfill these pivotal functions efficiently, K^+^ channels are found in virtually every cell of the human body, are distinguished by being the largest and most diverse class of ion channels, and are encoded by more than 70 genes (http://www.genenames.org/genefamily/kcn.php). An additional reason for their large diversity resides in the fact that they form macromolecular complexes, involving several proteins. The need for such a large number of K^+^ channels remains unclear.

In the past few years, several types of epilepsies have been associated to dysfunction of K^+^ channels, resulting from mutations in their encoding genes (Table 1), which appear prime elements potentially underlying idiopathic epilepsy. Indeed, the extremely high molecular and functional diversity of K^+^ channels, unmatched by any other types of channels, places them (*by statistical probability alone*) as primary targets of genetic variations. Besides the intrinsic K^+^ channels' gene defects associated with epilepsy, it is increasingly clear that also disruption/modification in K^+^ channel properties, even in the absence of a primary channel defect, may underlie increased susceptibility to seizures. The association between K^+^ channel dysfunctions and epileptic phenotypes is also confirmed by a multitude of animal models of epilepsy, that is, animals carrying K^+^ channel mutations or genetic manipulations and displaying spontaneous seizures or increased susceptibility to stimulus-induced seizure (Table 2). The extremely high diversity of K^+^ channels and the numerous variations identified in their genes often generate confusion in the classification of the associated diseases. Therefore, we propose to name the K^+^ channels defects underlying distinct epilepsies as “K^+^ channelepsies,” and offer a new classification according to a widely used K^+^ channel nomenclature (e.g., K_V_x.y). Moreover, here we discuss the different aspects of K^+^ channels dysfunctions underlying distinct epileptic phenotypes and describe the recent progress in the neurobiology of seizure susceptibility in animal models of epilepsy. Comprehensive knowledge of the neurobiological processes altered by K^+^ channel defects is a pivotal step to identify original therapeutic solutions for this devastating disease. Full understanding of how mutations in K^+^ channels give rise to distinct human and animal epileptic phenotypes requires a basic knowledge of their molecular features, expression pattern, and physiological roles. Thus, brief overviews on these topics for each K^+^ channel subfamily have been included.

**Table 1:**
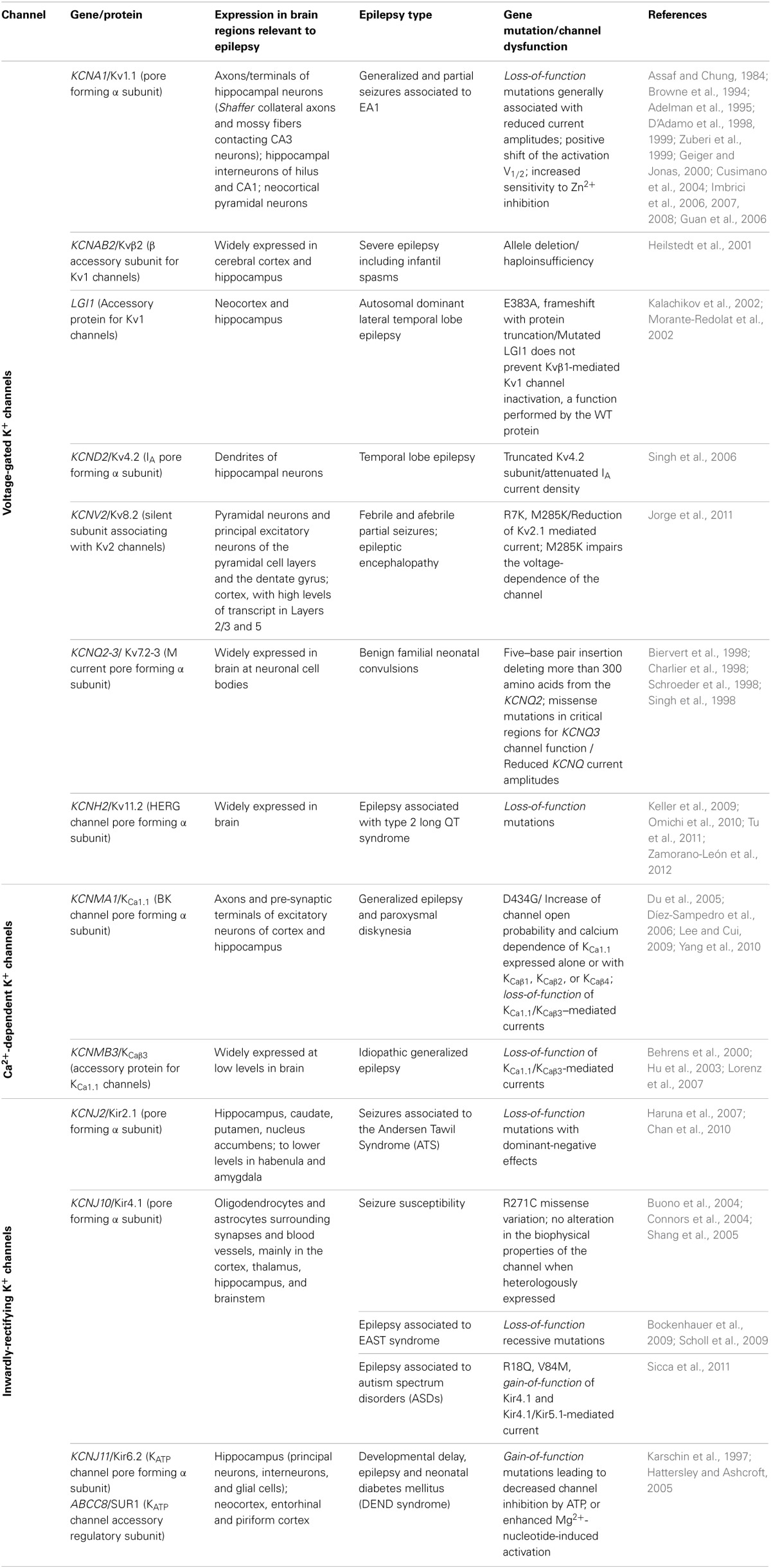
**Human K^+^ channelepsies**.

The table depicts the principal inherited human channelepsies caused by mutations in K^+^ channel α subunits or associated accessory proteins. For each channelepsy, information about the name of the mutated gene/protein, its expression in brain regions relevant to the pathology, type of epilepsy and association with other known syndromes, and dysfunction in channel behavior caused by the mutations are reported.

**Table 2:**
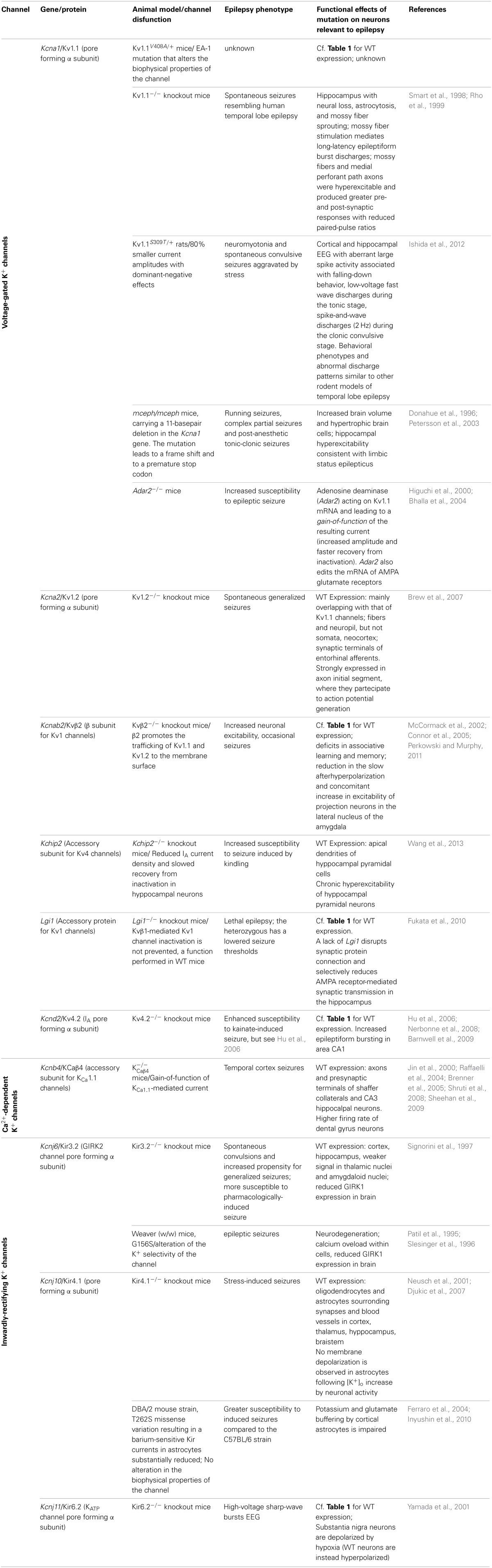
**Animal models of K^+^ channelepsies**.

The table depicts the principal animal models of epilepsy caused by mutations in K^+^ channel α subunits or associated accessory proteins. For each animal model, information about the name of the mutated gene/protein, type of epileptic phenotype, dysfunction in channel behavior caused by the mutation, and functional effects of the mutation are reported.

## Voltage-gated K^+^ channels and channelepsy

Voltage-gated K^+^ channels (Kv) are generally closed at the resting membrane potential of nerve cells (ca.–70 mV) and open following membrane depolarization. At a single channel level, membrane depolarizations elicit channel opening and closing (a process named *gating*) visible as upwards and downwards deflections of current trace (Pessia, 2004). The first Kv channel was cloned from the *Shaker* mutant of *Drosophila melanogaster* in 1987 (Tempel et al., 1987). The human ortholog of *Shaker* K^+^ channel is encoded by the gene *KCNA1*(Kv1.1). Since the first cloning, several other genes encoding for Kv channels have been identified from many different species. Based on sequence relatedness, Kv channels have been classified in subfamilies by using the abbreviation *Kvy.x* (Chandy and Gutman, 1993). According to this standardized nomenclature *Shaker*-related channels have been classified in the subfamily *Kv1.x* and each member numbered Kv1.1 through Kv1.8. The same criteria have been used to classify channels related to the *Drosophila* subfamilies *Shab* (Kv2.1 and Kv2.2), *Shaw* (Kv3.1 to Kv3.4), and *Shal* (Kv4.1 to Kv4.3). These channels may exist as homomers, whenever four identical α-subunits are assembled. However, different types of α-subunits may heteropolymerize to form channels with functional and pharmacological properties that are different from the parental homomeric channels (Isacoff et al., 1990; Ruppersberg et al., 1990).

The predicted 496 amino acids of the Kv1.1 α subunit form six transmembrane segments (TM) with the N- and C-termini residing inside the cell. TM5, TM6, and the H5 loop linking them contribute to the ion-conducting pore, and the *GYG* residues, that reside within the loop, control the K^+^ selectivity of the channel. The TM4 segment of each Kv1.1 α subunit is made of regularly spaced positively charged arginines and lysines and embodies the main voltage-sensor region that opens the channel by undergoing a conformational rearrangement upon membrane depolarization (Pessia, 2004). The full crystal structure, provided for a Kv channel, confirmed that this channel is composed of four homologous pore-forming α subunits (Jiang et al., 2003a,b). The description of the membrane-delimited Kv channel structure, T1 domain and β subunits allowed elucidation of many biophysical mechanisms controlling channel function. The Kv1 family members exhibit diverse expression patterns in the central and peripheral nervous system and are found tightly clustered within distinct neuronal compartments (Trimmer and Rhodes, 2004). Kv channels regulate the duration of action potentials, modulate the release of neurotransmitters, control the excitability, electrical properties, and firing pattern of central and peripheral neurons (Pessia, 2004). Moreover, the activity of Kv channels can be dynamically modulated by several events, including neurotransmitter- stimulated biochemical cascades (Imbrici et al., 2000; D'Adamo et al., 2013). Knowledge of their precise targeting and neurophysiological functions has important implications for defining the roles played by each Kv channel type in the pathophysiology of epilepsy.

## Kv1.1 channelepsy

Episodic ataxia type 1 (EA1) [OMIM 160120] is a *Shaker*-like K^+^ channels disease characterized by constant myokymia and dramatic episodes of spastic contractions of the skeletal muscles of the head, arms, and legs with loss of both motor coordination and balance (D'Adamo et al., 2012). EA1 was clearly described during the mid '70s by van Dyke and colleagues who first reported electroencephalographic (EEG) recordings characterized by runs of paroxysmal slow waves and generalized motor seizures in the proband's mother (van Dyke et al., 1975). The subsequent genetic analysis revealed that the individuals displaying epilepsy carried the F184C mutation in their *KCNA1* gene that profoundly altered the channel's properties (Browne et al., 1994; Adelman et al., 1995). Confirmations of an increased susceptibility to seizures in EA1 came from several subsequent studies (Table 1). Isolated photosensitive generalized tonic–clonic seizure (Imbrici et al., 2008) and abnormal EEGs have been observed in individuals with EA1 (Zuberi et al., 1999). EEGs may be characterized by intermittent and generalized slow activity, frequently intermingled with spikes. Zuberi et al. (1999) described a 3-year-old boy who presented with an ictal EEG with rhythmical slow-wave activity over the right hemisphere, becoming spike-and-wave complexes that then spread to the left hemisphere. Neuronal circuit dysfunctions within the hippocampus have been postulated to play a role in seizures and cognitive dysfunctions associated with EA1. Indeed, the hippocampus is a major brain region of the limbic system which plays an important role in the consolidation of information and in spatial memory, and it is often the focus of epileptic seizures. In rodent hippocampus, Kv1.1, Kv1.2, and Kv1.4 are found in *Schaffer* collateral axons and are highly expressed in axons and terminals of the medial perforant path in the middle third of the molecular layer of the dentate gyrus. In particular, Kv1.1, Kv1.4, and Kvβ1.1 subunits are expressed in mossy fiber boutons (*swellings of mossy fiber axons*) that form *en passant* synapses with pyramidal neurons in CA3. The macromolecular channel complex, composed of these subunits, regulates the activity-dependent spike broadening of hippocampal mossy fiber boutons and, consequently, the amount of neurotransmitter released during high-frequency stimuli (Geiger and Jonas, 2000). Mutations associated with EA1 profoundly alter the function of heteromeric channels composed of Kv1.1, Kv1.2, Kv1.4, and Kvβ1.1 subunits (D'Adamo et al., 1999; Imbrici et al., 2006) that likely contributes to seizures susceptibility and cognitive symptoms (Imbrici et al., 2006) in EA1. Notably, epileptiform brain activity has also been associated with intracranial administration of Zn^2+^ salts, and changes in Zn^2+^ modulation of GABA receptors have been implicated in the etiology of epilepsy. Zn^2+^ is released from mossy fiber terminals in the hippocampus, and from the basket cell terminals of the cerebellum (Assaf and Chung, 1984) where Kv1 channel activity likely is subjected to Zn^2+^ modulation. Indeed, homomeric and heteromeric channels containing Kv1 subunits are inhibited by extracellular Zn^2+^, and a distinct EA1 mutation increases several folds the Zn^2+^ sensitivity of these channels (Cusimano et al., 2004; Imbrici et al., 2007). Whether Zn^2+^ plays a role in triggering epilepsy-like symptoms in EA1 remains an intriguing hypothesis. A murine model that recapitulates the EA1 phenotype (*mKv1.1^V408A/+^*) has been generated by inserting in the mouse *Kcna1*(mKv1.1) a very conservative *valine* to *alanine* substitution (V408A) previously identified in patients (Herson et al., 2003; Brunetti et al., 2012, Table 2). Although data concerning the *mKv1.1^V408A/+^* are not yet available, the role of Kv1.1 channels in the neurobiology of epilepsy has been investigated by using Kv1.1 knockout mice (*mKv1.1^−/−^*). The hippocampus of these animals displays morphological characteristics typical of epilepsy, with neural loss, astrocytosis, and mossy fiber sprouting (Rho et al., 1999). Moreover, *mKv1.1^−/−^* also exhibits frequent spontaneous seizures throughout adult life, although the intrinsic passive properties of CA3 pyramidal cells are normal (Table 2). Antidromic action potentials were recruited at lower thresholds in *mKv1.1^−/−^* slices, and mossy fiber stimulation triggered synaptically-mediated long-latency epileptiform burst discharges. These data indicate that loss of Kv1.1 results in increased excitability in the CA3 recurrent axon collateral system, perhaps contributing to the limbic and tonic–clonic components of the observed epileptic phenotype of EA1 (Smart et al., 1998). Recently, *in vitro* extracellular recordings were performed by using a multielectrode array to characterize spontaneous sharp waves and high frequency oscillations in *mKv1.1^−/−^* hippocampi. This study showed that the mossy fibers and medial perforant path axons of *mKv1.1^−/−^* were hyperexcitable and produced greater pre- and post-synaptic responses with reduced paired-pulse ratios. Microdissection of mossy fibers and perforant path in *mKv1.1^−/−^* hippocampal slices ameliorated the abnormal oscillatory pattern and improved spike timing. In contrast, blockade of Kv1.1 channels with *dendrotoxin-K* reproduced these effects in WT slices. These findings suggest that loss of Kv1.1 enhances synaptic neurotransmitter release in the CA3 region, which reduces spike timing precision of individual neurons, leading to disorganization of network oscillatory activity and promoting the emergence of fast ripples (Simeone et al., 2013).

N-ethyl-N-nitrosourea (ENU) mutagenesis has been widely used to generate animal models of human diseases. An ENU-mutagenized rat strain has been recently generated and named “*autosomal dominant myokymia and seizures*” (ADMS) rats (Ishida et al., 2012). Genetic analysis of these animals resulted in the identification of the missense mutation S309T in the voltage-sensor domain of Kv1.1 channels (*rKv1.1^S309T/+^*). This heterozygous mutation resulted in 80% smaller current amplitudes with dominant-negative effects (Table 2). From 16 weeks of age severe periodic seizures were observed, and by 30 weeks of age, 84% of *rKv1.1^S309T/+^* had died. Cooling induced severe neuromyotonia, ataxia and aberrant spike-and-wave discharges (2–3 Hz) associated with clonus behaviors. *S*pontaneous convulsive seizures from 10 to 16 weeks of age were aggravated by stress (*cage changing or animal handling*). Cortical and hippocampal EEG recordings identified aberrant large spike activity associated with falling-down behavior, low-voltage fast wave discharges detected during the tonic stage, and spike-and-wave discharges (2 Hz) detected during the clonic convulsive stage (Ishida et al., 2012). The behavioral phenotypes and abnormal discharge patterns in *rKv1.1^S309T/+^* are similar to other rodent models of temporal lobe epilepsy (TLE). Carbamazepine (CBZ) administration ameliorated seizures. The videos related to this animal model of EA1 are available online at doi:10.1016/j.brainres.2011.11.023.

The megencephaly mice, *mceph/mceph*, are characterized by increased brain volume, hypertrophic brain cells and slight hippocampal astrocytosis. They display a complex behavioral phenotype including running seizures, complex partial seizures and postanaesthetic tonic-clonic seizures (Donahue et al., 1996, Table 2). Remarkably, an 11-basepair deletion in the *Kcna1* gene of *mceph/mceph* mice has been identified (Petersson et al., 2003). The mutation leads to a frame shift and a premature stop codon that was predicted to truncate the protein at amino acid 230 (out of 495). Therefore, *mceph/mceph* mice express Kv1.1 subunits lacking the last five TMs and the C-terminal domain: The absence of Kv1.1 channels with intact C-terminal domains was confirmed by Western blotting analysis of whole protein extracts from brain. Electrophysiological investigations from *mceph/mceph* brain slices revealed hippocampal *mceph/mceph* brain slices revealed hippocampal hyperexcitability consistent with limbic status epilepticus (SE) C-teminal domain of Kv1.1 channels (R417stop) has also been found in a EA1 proband displaying episodes of ataxia precipitated by exercise, stress, startle or high temperature occurring after a hot bath or when using a hairdryer but, absence of epilepsy (Eunson et al., 2000).

Kv1.2 knockout mice display increased seizure susceptibility (Brew et al., 2007, Table 2). It should be recalled that Kv1.1 and Kv1.2 are closely coupled K^+^ channel subunits, as they form heteromeric channels in several brain regions. Indeed, biochemical and electrophysiological studies have shown that Kv1.1/Kv1.2 channels control neuronal excitability, action potentials propagation and synaptic transmission. Notably, EA1 mutations alter the function of heteromeric channels composed of Kv1.1 and Kv1.2 subunits (D'Adamo et al., 1999). In conclusion, these investigations with animal models of Kv1.1 channelepsy highlighted the crucial brain regions that are likely the site of origin of abnormal discharges in EA1 and the relevant mechanisms underlying their susceptibility to seizures.

## Role of RNA editing in Kv1.1 channelepsy

Kv1.1 mRNA is target for enzymatic deamination by adenosine deaminase acting on RNA (ADAR2). Knockout mice for the ADAR2 gene are prone to epileptic seizures and die within a few weeks after birth (Higuchi et al., 2000, Table 2). Kv1.1 editing by ADAR2 results in channels with an I400V exchange in the S6 segment (*Kv1.1^I400V^*). *In vitro*, an increase in increase in *Kv1.1^I400V^* editing increases K^+^ outward current upon membrane depolarization and accelerates recovery from inactivation at negative membrane potentials (Bhalla et al., 2004). Interestingly, increased levels of *Kv1.1^I400V^* editing were found in chronic epileptic rats. It has also been reported a reduced ability of 4-AP to trigger seizure-like effects in brain slices dissected from a kainic acid rat model of chronic epilepsy (Zahn et al., 2008; Streit et al., 2011). A similar phenomenon was observed in human brain slices of patients with pharmacoresistant TLE (Gabriel et al., 2004). The postulated ictiogenic mechanism of 4-AP action is its ability to block *Kv1* and *Kv3* channels, which results in increased transmitter release. *Kv1.1^I400V^* editing results in 4-AP–insensitive homomeric *Kv1.1* channels, and alters the pharmacology of heteromeric channels (Decher et al., 2010; Streit et al., 2011). These findings suggest that mRNA editing of Kv1.1 in chronic epilepsy may to the reorganization of the entorhinal cortex and have anticonvulsive effects. The editing of Kv1.1 mRNA could therefore represent a compensatory mechanism in brain areas where epileptic seizures originate. TLE, namely spontaneous seizures involving the hippocampal formation, is the most prevalent refractory epilepsy. TLE can be sub-classified into mesial, lateral and neocortical. Mesial TLE (MTLE) is defined by clinic-anatomical observations and characterized by seizures arising from the hippocampus or parahippocampal structures, and frequent pharmacotherapy resistance. A recent screening study identified a significant negative correlation between epilepsy duration in patients with MTLE plus hippocampal sclerosis and the I/V editing site of Kv1.1 channels. This may be the result of the epileptic process itself, together with the medication history of the patient, and may thus reflect compensatory mechanisms to either of these factors (Krestel et al., 2013).

## Kv4 channelepsy

Kv4 channels underlie the main dendritic A-type Kv currents in hippocampal neurons and play a critical role in regulating the extent to which back-propagating action potentials invade the dendritic tree. They also impact the propagation of synaptic potentials from the dendritic arbor to the soma (Jerng et al., 2004). The relevance of Kv4.2 to epilepsy comes from the identification of a mutation in a patient with TLE, which results in the expression of a truncated Kv4.2 subunit (Singh et al., 2006, Table 1), and the observation that pharmacological blockade of Kv4 channels is epileptogenic (Avoli, 2001). These studies support the concept that Kv4.2 deficiency may contribute to aberrant network excitability and regulate seizure threshold. Intriguingly, it has been reported that Kv4.2 knockout mice do not exhibit an overt seizure phenotype (Hu et al., 2006), perhaps due to posttranslational *up-regulation* of other Kv currents (Nerbonne et al., 2008). A more recent study, however, suggests that loss of Kv4.2 channels is associated with enhanced susceptibility to seizures after kainate injection (Barnwell et al., 2009, Table 2).

Systemic administration of the muscarinic agonist pilocarpine to rats recapitulates the features of human limbic seizures and SE. The expression of a number of different K^+^ channels, including Kv (Monaghan et al., 2008) and K_Ca_ (BK) channels (Pacheco Otalora et al., 2008) is altered in response to pilocarpine-induced SE. The amplitude of A-type currents in the dendrites of hippocampal CA1 pyramidal neurons is reduced in response to pilocarpine-induced seizures (Bernard et al., 2004). In addition to Kv4.2, Kv4.3 and KChIP2 current decrease, pilocarpine-treated rats exhibited staining changes for these proteins in the molecular layer of the dentate gyrus from being uniformly distributed across the molecular layer to become concentrated in just the outer two-thirds (Monaghan et al., 2008). As a consequence, an increased dendritic excitability was found in CA1 pyramidal cell dendrites in response to the same pilocarpine model of TLE (Bernard et al., 2004). *In situ* hybridization studies revealed that generalized seizures induced by pentylenetetrazol (Tsaur et al., 1992) or kainic acid (Francis et al., 1997) also altered the regional hippocampal gene expression of rat Kv4.2. A decrease of A-current density in this proximal region of the granule cell dendrite—a location that receives massive aberrant excitatory mossy fiber input following induction of SE and as spontaneous seizures develop—would lower the firing threshold and thus contribute to the development of spontaneous recurrent seizures. On the other hand, seizures *in vivo* and glutamate *in vitro* induce a rapid surface recruitment of Kv4.2 channels in neurons. Thus, seizure would induce dampening of phasic firing, generated by glutamatergic synaptic transmission, by enhancing the surface expression of Kv4.2 channels. Interestingly, mutant LGI1 blocks this homeostatic neuronal response (Smith et al., 2012). Thus, it seems that convulsants may affect Kv4 currents in different ways and compensatory pathways may be recruited to dampen hyper-excitability.

One of the most pronounced anatomical effects of TLE, exhibited in the pilocarpine animal model, is mossy fiber sprouting. The expansion of Kv1.4 staining in stratum lucidum of CA3 in pilocarpine-SE animals suggests that expansion of the mossy fiber terminal field, due to SE-induced sprouting, is accompanied by parallel increases in targeting of Kv1.4 to the newly sprouted mossy fiber axons and terminals. Changes in Kv1.4 immunoreactivity were observed also in the molecular layer of the dentate gyrus. In conclusion, variations in A-type Kv1.4-containing channels in mossy fiber presynaptic terminals, and Kv4.2-containing channels in dentate granule cell and CA1 dendrites, represent important mechanisms intervening in the acquisition of the permanent epileptic phenotype in this animal model of human TLE and attractive therapeutic targets. Moreover, these studies highlighted the essential nature of Kv4.2 and the specific contributions of its auxiliary KChIP subunits (*see below*) in regulating the seizure susceptibility associated with epileptogenesis.

## Kv7-M-channelepsy

Five members of the *KCNQ* gene family have been identified (*KCNQ1-5*) which form homomeric or heteromeric K^+^ channels. In many brain regions, heteromeric *KCNQ2*(Kv7.2)/*KCNQ3*(Kv7.3) channels seem the major determinant of “*M*” currents that are inhibited by several neurotransmitters, including acetylcholine (ACh) through the muscarinic receptors (Devaux et al., 2004; Pan et al., 2006). Functionally, these slow-gating K^+^ channels that are open at subthreshold voltages, contribute to setting the resting membrane potential of neurons, prevent repetitive firing, and control spike-frequency adaptation. Indeed, blockade of M-currents is associated with depolarization of the resting membrane potential, and generation of trains of action potentials (Brown and Adams, 1980; Delmas and Brown, 2005). Mutations in *KCNQ2* or *KCNQ3* cause a form of juvenile epilepsy called *benign familial neonatal convulsions* (Biervert et al., 1998; Charlier et al., 1998; Schroeder et al., 1998; Singh et al., 1998, Table 1). The clinical features of *KCNQ2*-related benign familial neonatal epilepsy (*KCNQ2-BFNE*) are characterized by tonic or apneic episodes, focal clonic activity, or autonomic changes that start between the second and eighth day of life and spontaneously disappear between the first and the sixth to 12^th^ month. The *KCNQ2*-related epileptic encephalopathy (*KCNQ2-NEE*) is characterized by multiple daily seizures that begin the first week of life, are mostly tonic, with motor and autonomic features, and cease after 9 months to 4 years. Most affected individuals are intellectually impaired (Bellini et al., 1993-2013). Just a 25% decrease in M-current amplitude is sufficient to drive human neurons to epileptogenic levels, and causes neonatal epilepsy (Schroeder et al., 1998). The clinical severity of the disease may be related to the extent of mutation-induced functional K^+^ channel impairment (Miceli et al., 2013). Drugs that enhance neuronal M-currents, such as retigabine, represent a valuable therapeutic treatment for certain hyperexcitatory diseases, including epilepsy (Porter et al., 2012). Flupirtine, a structural analogue of retigabine, which also activates channels formed by neuronal Kv7 subunits, has been shown to be effective in animal models of neonatal convulsion (Raol et al., 2009). Kv7.1 (*KCNQ1*) channels are mainly expressed in the heart where they contribute to termination of the action potential. Mutations in *KCNQ1*(Kv7.1) are responsible for one form of long QT syndrome (LQT1) (Wang et al., 1996). However, Kv7.1 channels are also expressed in the brain. Intriguingly, LQT1 patients exhibit an increased risk of epilepsy, suggesting a relationship between these two diseases (*see below*). Notably, M-channels are also highly sensitive to intracellular Ca^2+^ variations, being inhibited by Ca^2+^ with an IC50 of ~100 nM in sympathetic neurons (Selyanko and Brown, 1996; Gamper and Shapiro, 2003). Recently, it has been proposed that abnormal [Ca^2+^]i transients induced by Kv1.1 channel dysfunction may result in repetitive discharges in myelinated nerves (Brunetti et al., 2012). This mechanism may underlie not only the neuromyotonic/myokymic discharges typically observed in EA1 individuals, but also their susceptibility to seizures.

The neuronal serum- and glucocorticoid-regulated kinase 1 (SGK1.1) appears to be a physiological M-current regulator, as it enhances Kv7.2/3 current levels. Transgenic mice expressing a constitutively active form of SGK1.1 are resistant to kainic acid-induced seizures which occur mainly in the temporal lobe. These findings indicate that SGK1.1 activity can regulate neuronal excitability through M-current modulation and protects against seizures (Miranda et al., 2013).

## Kv8 channelepsy: role of the silent modifiers of K^+^ channels in epilepsy

A number of subunits have been cloned and classified in the K^+^ channel subfamilies Kv6, Kv8, and Kv9. Although they possess the hallmarks of Kv subunits they do not appear to form functional homomeric K^+^ channels when expressed alone and, therefore, have been named “*silent*” subunits. However, these proteins do co-assemble with other Kv subunits (e.g., Kv2), and confer distinct biophysical properties to heteromeric channels Kv2/Kv6, Kv2/Kv8, or Kv2/Kv9. Thus, these gene products are also known as “*silent modifiers*” of Kv channels. In particular, Kv8.2 (*KCNV2*) co-assembles with Kv2.1 as a heterotetramer, significantly reducing the surface expression of the resulting channels and influencing their biophysical properties. Within the hippocampus, Kv2.1 and Kv8.2 co-localize in pyramidal neurons and in the principal excitatory neurons of the pyramidal cell layers and the dentate gyrus. Moreover, both are expressed in the cortex, with high levels of transcript in layers 2/3 and 5 (Allen brain atlas; http://www.brain-map.org). These regions are critically involved in seizure generation and propagation. It has been shown that Kv2.1 channels contribute significantly to the delayed-rectifier K^+^ currents in hippocampal neurons (Murakoshi and Trimmer, 1999). Importantly, knockdown of Kv2.1 in hippocampal slices resulted in increased CA1 pyramidal neuron excitability under conditions of high-frequency stimulation (Du et al., 2000). Thus, the Kv2.1 current reduction, mediated by the inclusion of Kv8.2 subunits in heterotetramers, could regulate the membrane repolarization and excitability of hippocampal neurons, contributing to seizure susceptibility. Indeed, mutations in two related silent subunits have been associated with neurological disorders (Table 1). In particular Kv8.2 (*KCNV2*) with epilepsy (Jorge et al., 2011) and Kv9.1 (*KCNS1*) with chronic pain (Costigan et al., 2010). Recently, an individual affected by febrile and afebrile partial seizures has been reported to carry a genetic variation in *KCNV2*, inherited from his unaffected mother. This resulted in the substitution of a highly conserved lysine for an arginine (R7K) in the cytoplasmic amino terminus of Kv8.2 subunits. An additional patient who inherited a methionine to arginine mutation (M285R) in Kv8.2 subunits from his unaffected mother was likewise identified with an epileptic encephalopathy and with severe refractory epilepsy (Jorge et al., 2011). The pathogenic relevance of these variants was assessed by electrophysiological recordings from cells co-expressing the human Kv2.1 with Kv8.2 wild-type or its mutated subunit (R7K or M285R). This assay showed that both variants suppressed Kv2.1-mediated current more than the wild-type; in addition, the M285R impaired the voltage-dependence of the channel. These results suggest that both variants enhance seizure susceptibility of affected patients by reducing neuronal delayed-rectifier K^+^ channel function in brain regions critically involved in seizure generation and propagation (Jorge et al., 2011).

The severity of seizures in mouse models of epilepsy is highly dependent on their genetic background. In transgenic mouse models of sodium channel-dependent epilepsy, the phenotype is more severe in SJL/J compared with the C57BL/6J strain (Bergren et al., 2005). Interestingly, it has been shown that the hippocampal Kv8.2 (*Kcnv2*) transcript is ~3-fold greater in SJL/J compared with the C57BL/6J strain (Jorge et al., 2011). The enhanced availability of Kv8.2 subunits for co-assembling with Kv2.1 would remarkably reduce the surface expression of the resulting channels in hippocampal neurons of SJL/J mice contributing to their greater susceptibility to seizures.

In conclusion, these studies implicate Kv8.2 (*Kcnv2*) as an epilepsy gene in rodents and humans. Moreover, although silent subunits have been mostly ignored since they were first cloned a decade or more ago, their potential clinical relevance is now becoming fully evident and therefore represents an attractive new avenue of investigation in neurologic channelopathies research.

## Kv11-HERG-channelepsy

The human ether-a-go-go-related gene (HERG) encodes for voltage-gated K^+^ channels. HERG channels exhibit functional properties remarkably different from other K^+^ channels (Tristani-Firouzi and Sanguinetti, 2003). They are widely expressed in the brain where they contribute to setting the frequency and the discharge stability of neurons, and to adapting their intrinsic properties to signal processing (Pessia et al., 2008). They also modulate the excitability of dopaminergic and GABAergic neurons (Nedergaard, 2004; Canavier et al., 2007). HERG channels are expressed in the heart where they control the repolarization of ventricular action potentials. *Loss-of-function* mutations in *KCNH2* (HERG) cause type 2 long QT syndrome (LQT2), a condition in which the induced delayed repolarization of the heart following a heartbeat increases the risk of episodes of sudden death due to ventricular fibrillation. However, LQT syndrome is closely associated with seizure and frequently it is misdiagnosed as epilepsy (Table 1). Sudden unexpected death in epilepsy is the most frequent epilepsy-related cause of death for which an underlying arrhythmogenic predisposition has been suggested. Several clinical reports have recently described seizures and arrhythmic events in LQT2 triggered by visual or acoustic stimuli (Keller et al., 2009; Omichi et al., 2010; Tu et al., 2011; Zamorano-León et al., 2012). Considering that HERG channels control several neuronal electrical features, including discharge dynamics (Pessia et al., 2008), these clinical findings raise the possibility that alteration in *KCNH2*-encoded K^+^ channels may confer susceptibility for epilepsy and cardiac LQT2 arrhythmia.

## Auxiliary subunits of K^+^ channels and channelepsies

K^+^ channels expressed by distinct cell types may be formed by unique hetero-oligomeric complexes comprising auxiliary subunits. Several types of these subunits have been identified including beta-subunits (Kvβ), minK (minimal K^+^ channel peptide), MiRP (minK-related peptide), KChAP (K^+^ channel-associated protein), KChIP (K^+^ channel-interacting protein) and neuronal calcium sensor (NCS). Each type of auxiliary subunit modulates the activity of the associated K^+^ channel in distinct ways. Defects in these subunits may alter the function of the channel and result in increased seizure susceptibility.

## Kvβ channelepsy

Auxiliary subunits such as Kvβ 1.1 and Kvβ 1.2 confer fast *N-type* or *A-type* inactivation to non-inactivating Kv1 channels by means of a “*ball-and-chain*” mechanism of pore occlusion whereby the tethered (*chain*) positively charged inactivation particle (*ball*) in the amino-terminus of the Kvβ 1 subunit binds to the intracellular entrance of the pore discontinuing the outflow of K^+^ ions. The fast inactivation of delayed-rectifier K^+^ channels is a physiologically relevant process, as it controls the firing properties of neurons and their response to input stimuli (Pessia, 2004). Surprisingly, Kvβ 1.1 knockout mice do not display an overt epileptic phenotype. On the other hand, Kvβ 2 knockout and Kvβ 1/Kvβ 2 double-knockout mice are characterized by an increased neuronal excitability, occasional seizures, cold swim-induced tremors and a reduced life span (McCormack et al., 2002; Connor et al., 2005, Table 2). Clinical investigations have found an association between the severity of seizures, including infantile spasms and the loss of the Kvβ 2 gene. Moreover, the hemizygosity of this gene in epileptic patients suggests that haploinsufficiency for *KCNAB2* is a significant risk factor for epilepsy (Heilstedt et al., 2001). Notably, Kvβ 2 subunits do not confer fast inactivation properties to Kv1 channels, since they lack the inactivation particles. Thus, a chaperone-like function has been proposed for these subunits. Although these findings are consistent with a role for accessory subunits in regulating central nervous system excitability, further functional assays are necessary to determine thoroughly how loss or haploinsufficiency of the Kvβ 2 gene affects distinct network excitability and causes Kvβ 2 channelepsy.

## Kv_LGI1_ channelepsy

The *leucine-rich glioma-inactivated-1* (*LGI1*) is a secreted neuronal protein, complexed with Kv channels, and highly expressed in neocortex and hippocampus. *LGI1* mutations—e.g., *the point mutation E383A that prevents the neuronal secretion of LGI1*—have been found in patients with autosomal dominant lateral temporal lobe epilepsy (ADLTE), a syndrome characterized by partial seizures with acoustic or other sensory hallucinations (Kalachikov et al., 2002; Morante-Redolat et al., 2002; Fukata et al., 2010, Table 1). Moreover, loss of *Lgi1* in mice causes lethal epilepsy (Fukata et al., 2010, Table 2). In the hippocampus, both Kv1.1 and *Lgi1* appear co-assembled with Kv1.4 and Kvβ 1 in axonal terminals. In A-type channels composed of these subunits, *Lgi1* prevents N-type inactivation mediated by the Kvβ 1 subunit. In contrast, defective *LGI1* molecules identified in ADLTE patients fail to exert this effect, which results in channels with rapid inactivation kinetics. These data suggest that these changes in inactivation gating of presynaptic A-type channels may promote epileptic activity (Schulte et al., 2006).

Specific auto-antibodies underlie an emerging class of seizures named “autoimmune epilepsy” that involves K^+^ channels. Indeed, several of these auto-antibodies do not bind directly with Kv1.1, Kv1.2, or Kv1.6 channels, as previously believed, but rather to associated proteins such as *LGI1*, contactin-associated protein 2 (*CASPR2*), contactin-2, or others to be identified (Irani et al., 2010; Lai et al., 2010; Lancaster et al., 2011).

## Kv_KCHIP1_ channelepsy

Cytosolic Kv channel-interacting proteins KChIP1–KChIP4 (An et al., 2000), which belong to the NCS family of calcium binding EF-hand proteins, co-assemble with the N-terminus of Kv4 subunits (Zhou et al., 2004) to form a native complex that encodes major components of neuronal somatodendritic A-type K^+^ current (*I*_A_). KChIP2 expression is high in the hippocampus, particularly within the apical dendrites of pyramidal cells. *KChIP2* gene deletion in mice (*Kchip2*^−/−^) affected the *I*_A_ in hippocampus, namely reduced current density, decreased channel availability, and slowed recovery from inactivation. This results in chronic hyper-excitability in hippocampal pyramidal neurons and *Kchip2*^−/−^ mice exhibited increased susceptibility to seizures induced by kindling (Table 2). However, a compensatory up-regulation of inhibitory synaptic activity (*up-regulation of GABA currents*) was also observed (Wang et al., 2013). These findings indicated that *KChIP2* is essential for homeostasis in hippocampal neurons and mutations in these K^+^ channel auxiliary subunits may be loci for epilepsy (Wang et al., 2013). Interestingly, *Kchip2*^−/−^ mice are highly susceptible to cardiac arrhythmias (Kuo et al., 2001). Thus, this evidence suggests that *loss-of-function* mutations in *KChIP2* could confer an increased susceptibility to both seizures and cardiac arrhythmias, increasing the risk to sudden unexpected death in epileptic patients.

In conclusion, these studies demonstrated that the auxiliary subunits play important roles in the pathogenesis of epilepsy by affecting K^+^ channel function and network excitability in distinct ways (*see also K*_Ca1.1_ *channelepsy*).

## Calcium-activated K^+^ channels and channelepsy

The calcium-activated K^+^ (K_Ca_) channels are highly conserved across species, and widely expressed in the human brain. The phylogenetic tree of the K_Ca_ channels shows that they are made of two genetically well-distinct groups (Wei et al., 2005), the large conductance (BK; K_Ca1.1_), and the small/intermediate-conductance (SK/IK; K_Ca2.1_, K_Ca2.2_, K_Ca2.3_, K_Ca3.1_) K_Ca_ channels. With regard to gating mechanism, the Ca^2+^ sensitivity of SK/IK channels is provided by tightly bound calmodulin (Xia et al., 1998; Fanger et al., 1999), in contrast to the direct binding of Ca^2+^ at specific internal sites on the channel protein of K_Ca1.1_ channels (Lee and Cui, 2010). Moreover, unlike the SK/IK channels, K_Ca1.1_ channels are also activated by voltage.

In brain neurons K_Ca_ channels are widely distributed in the axons plasma membrane and at the presynaptic terminals (Knaus et al., 1996; Blank et al., 2004), and often located close to voltage-gated Ca^2+^ channels (Ca_*v*_; Marrion and Tavalin, 1998). The Ca^2+^ influx that follows neuronal excitation activates K_Ca_ channels whose outward K^+^ flux contributes to terminate the action potential and establish the afterhyperpolarization (AHP) that closes Ca_v_ channels. This negative feedback control has been generally assumed to make K_Ca_ channels critical players in opposing repetitive firing and hyperexcitability typical of epileptic disorders. To date only mutations in the K_Ca1.1_ channel have been clearly associated to epilepsy.

## K_Ca1.1_ channelepsy

The K_Ca1.1_ channel, originally cloned from the *Drosophila slowpoke* locus, thus the name *slo* (Atkinson et al., 1991), is coded by one single gene, the *KCNMA1* (Wei et al., 2005). The variety observed in biophysical and pharmacologic properties of the channel derives from the extensive alternative splicing of its mRNA (Tseng-Crank et al., 1994), and the type of accessory β subunit (K_Caβ_) that associates with the channel (Jiang et al., 1999). The K_Ca1.1_ channel is composed of four identical pore-forming α subunits, each displaying a transmembrane portion very much like the Kv channel, with the voltage sensing domain made by segments S1–S4, and the permeating pore domain by the S5, P, and S6 segments. Unlike Kv channels, the K_Ca1.1_ channel has an additional transmembrane domain (S0), thought to subserve for (K_Caβ_) subunit interaction and modulation of the channel (Morrow et al., 2006). The channel has also a large C-terminal cytosolic domain that confers Ca^2+^ sensitivity to the channel. Notably, the amino-acid sequence of the C-terminal domain contains no conventional Ca^2+^ binding motif such as EF hands or C2 domains. The two putative high affinity Ca^2+^ binding sites of the channel are formed, respectively, by two closely located (five amino acids apart) aspartate residues (Xia et al., 2002) on the RCK1 (regulator of K^+^ conductance) domain, and by a series of aspartate residues in a region known as the Ca^2+^ bowl, located in the RCK2 domain (Wei et al., 1994; Schreiber and Salkoff, 1997).

K_Ca1.1_ channels expression predominates in axons and pre-synaptic terminals of excitatory neurons located in epileptic relevant structures, such as cortex and hippocampus (Knaus et al., 1996; Hu et al., 2001; Misonou et al., 2006; Martire et al., 2010). In brain neurons, the Ca^2+^ that activates K_Ca1.1_ channels enters primarily through Ca_v_ channels (Berkefeld et al., 2006), with which K_Ca1.1_ channels strictly co-localize in order to be activated during an action potential by the Ca^2+^ microdomains that form around the Ca^2+^ source (Müller et al., 2007). The K_Ca1.1_ channel activity is thus limited by the duration of the action potential-evoked Ca^2+^ transients, and consequently restricted to the action potential repolarization phase and the fast portion of the after-hyperpolarization (fAHP; Sah and Faber, 2002), and generally assumed to reduce neuronal excitability. Recent findings point, however, to a role of K_Ca1.1_ channels in promoting high frequency firing, an effect likely attributed to fast spike repolarization, fAHP generation and the consequent reduction in the activation of other slower Kv channels and of the inactivation of Na^+^ channels (Storm, 1987a,b; Gu et al., 2007). Whereas they appear to be virtually uninfluential to synaptic release modulation under physiologic conditions (Hu et al., 2001; Raffaelli et al., 2004; Shruti et al., 2008; Martire et al., 2010).

Given the role of K_Ca1.1_ channels in promoting high neuronal firing frequency and their predominant expression in excitatory neurons of cortex and hippocampus, it is no surprising that several lines of evidence from animal models point to a pro-epileptic role for K_Ca1.1_ channels. For example, spontaneous cortical bursting in mice with high susceptibility to convulsions was completely inhibited by the K_Ca1.1_ channel blocker *iberiotoxin* (IbTX) (Jin et al., 2000). A similar inhibitory effect of K_Ca1.1_ channel antagonists was reported on chemoconvulsant-induced seizures *in vitro* and *in vivo* (Jin et al., 2000; Shruti et al., 2008; Sheehan et al., 2009). Finally, K_Caβ4_ knockout mice displayed temporal cortex seizures and a *gain-of-function* of K_Ca1.1_ channels in dentate gyrus slices, resulting in higher firing rate (Brenner et al., 2005, Table 2).

Evidence for an association of human epilepsy with the K_Ca1.1_ channel has also been found. A missense mutation (D434G) in the *KCNMA1* gene coding for the α subunit has been found in family patients suffering from generalized epilepsy and paroxysmal diskinesia (Du et al., 2005, Table 1). Expression studies indicated that the D434G mutant channel displayed markedly greater macroscopic currents and single channel open probability, resulting from a 5-fold increase in Ca^2+^ sensitivity (Du et al., 2005). In accordance, the mutation appeared to be located in the RCK1 domain, close to the putative Ca^2+^ binding site and the segments subserving the allosteric coupling between Ca^2+^ binding site and the activation gate (Yang et al., 2010). Subsequent studies showed that the effects of the D434G mutation on the K_Ca1.1_ channel properties were similar or even more pronounced in the presence of the K_Caβ1_, K_Caβ2_, or K_Caβ4_ subunit, highly expressed in the brain (Díez-Sampedro et al., 2006; Lee and Cui, 2009). Notably, a polymorphism in the K_Caβ4_ subunit has been associated to human MTLE in an Irish cohort, but this has not been confirmed in other populations (Cavalleri et al., 2007; Manna et al., 2013). By contrast, the α subunit coexpressed with K_Caβ3b_ (a splicing variant of K_Caβ3_) was modified by the mutation with a slowing of the activation, a reduction in the voltage-dependence, but no change in Ca^2+^-dependence, suggesting a *loss-of-function* of the K_Ca1.1_ current (Lee and Cui, 2009). Interestingly, a K_Caβ3_ single-nucleotide mutation, causing a *loss-of-function* of K_Ca1.1_ current containing the K_Caβ3b_ subunit, displays a small but significant association with idiopathic generalized epilepsy (Hu et al., 2003; Lorenz et al., 2007, Table 1). Taken together, these data suggest that both a *loss-of-function* of K_Caβ3b_-containing K_Ca1.1_ channels and a *gain-of-function* of K_Caβ1_, K_Caβ2_, or K_Caβ4_-containing K_Ca1.1_ channels would favor the epileptic phenotype, but more information on the location and function of K_Caβ3b_ subunits is needed to clarify this point.

A modulation of K_Ca1.1_ channel expression in epilepsy models has also been found. A *gain-of-function* of K_Ca1.1_ currents associated with increased spontaneous and evoked firing rates occurs in mouse neocortical pyramidal neurons 24 h after chemoconvulsant-induced generalized tonic-clonic seizures (Shruti et al., 2008). Conversely, in a model of pilocarpine-induced MTLE K_Ca1.1_ channel α subunit was down-regulated at the protein and mRNA level in hyppocampal mossy fibers originating from the dentate gyrus (Ermolinsky et al., 2008; Miceli et al., 2013). Notably, K_Ca1.1_ channel proteins remaining after seizure induction were mostly changed to the STREX splicing isoform, displaying an increased Ca^2+^-sensitivity with respect to the ZERO splice variant normally present (Ermolinsky et al., 2008). The functional consequence of the observed changes is thus not clear.

## Inwardly-rectifying K^+^ channels and channelepsies

Members of the inwardly-rectifying family of K^+^ channels (Kir) are found in virtually every cell type where they are major regulators of K^+^ fluxes across membranes (Hibino et al., 2010). The principal role of most Kir channels is the maintenance of the resting membrane potential and thereby the control of cell excitability, while others subserve the transport and recycling of K^+^ across membranes. Like other K^+^ channels, Kir subunits assemble as tetramers, and their ability to heteromultimerise adds functional diversity to a limited number of gene products. Kir subunits possess two transmembrane domains and approximately 15 distinct Kir clones have been identified so far, forming seven major subfamilies: Kir1–Kir7 (Bond et al., 1994; Hibino et al., 2010). Important physiological roles have been established for nearly all of these subfamilies. Generally, a Kir channel acts as a diode whereby the inward current through these channels is greater at potentials more negative than the *E_K_*, as compared to more positive values, where the outward flow is inhibited and the membrane potential free to change. The rectifying nature of Kir channels is due to a voltage-dependent block of the intracellular side of the pore by cytoplasmic polyamines and Mg^2+^ ions (Matsuda et al., 1987; Lopatin et al., 1994; Lu and MacKinnon, 1994; Stanfield et al., 1994). Several studies highlighted the role of inwardly-rectifying K^+^ channels' dysfunction in neuropsychiatric disorders and epilepsy although the relevant mechanisms in some instances await clarification.

## Kir2 channelepsy

Kir2.1 channels are highly expressed in brain, particularly in hippocampus, caudate, putamen, nucleus accumbens, and to lower levels in habenula and amygdala (Karschin et al., 1996), where they contribute to control neuronal excitability. In particular, the amplitude of Kir2.1 currents is small in young dentate granule neurons (DGCs), and increases ~3-fold in mature DGCs to optimize their excitability. Thus, Kir2.1 channels play an important role in DGCs firing properties during development (Mongiat et al., 2009). Moreover, Kir2.1 channels in combination with Kir4.1 control the astrocyte-mediated K^+^ buffering (Bordey and Sontheimer, 1998; Jabs et al., 2008; Chever et al., 2010). It has been proposed that *up-regulation* of Kir2.1 in DGCs would counterbalance the hyper-excitability observed in TLE, thus functioning as an anti-convulsant (Young et al., 2009). Individuals harboring *loss-of-function* mutations in *KCNJ2* (e.g., Andersen-Tawil syndrome; OMIM 170390) may present with mood disorders and seizures (Haruna et al., 2007; Chan et al., 2010, Table 1), suggesting a possible role for Kir2.1 channels in the pathogenesis of neuropsychiatric disorders and epilepsy (Haruna et al., 2007; Chan et al., 2010).

## Kir3-GIRK-channelepsy

Several neurotransmitters, including dopamine, opioid, somatostatin, acetylcholine, serotonin, adenosine, and GABA exert their actions by modulating the activity of G protein-coupled Kir channels (GIRK) belonging to the subfamily 3 (Kir3). Four subunits have been cloned: GIRK1-GIRK4, also known as Kir3.1–Kir3.4, that may heteropolimerize. Generally, receptors activation of intracellular heterotrimeric G proteins αβγ leads to stimulation of heteromeric Kir3 channels activity, resulting in an outward flux of K^+^ ions that causes membrane hyperpolarization and inhibition of cell excitability (Krapivinsky et al., 1995; Slesinger et al., 1995; Tucker et al., 1996a). The crystal structure of this channel type has been resolved, recently (Whorton and Mackinnon, 2013). *Gain-of-function* of Kir3 channels can considerably reduce neuronal activity, whereas *loss-of-function* can lead to excessive neuronal excitability and epilepsy. Indeed, ablation of the gene encoding for Kir3.2 channels (GIRK2) results in spontaneous convulsions and increased susceptibility for generalized seizures in rodents (Signorini et al., 1997, Table 2). The principal phenotype of *weaver* mice (*wv/wv*) is an ataxic gait, due to severe hypoplasia of the cerebellum, learning deficits and epileptic seizures. *Weaver* mice carry a deleterious mutation in the pore of Kir3.2 channels (G156S; Patil et al., 1995, Table 2). This mutation alters the K^+^ selectivity of the channel, induces calcium overload in cells, and reduces channel availability (Slesinger et al., 1996; Tucker et al., 1996b) with a mechanism different from heteromeric subunit degradation (Tucker et al., 1996c). It is likely that these molecular defects, induced by the G156S mutation, would lead to neurodegeneration and seizures susceptibility that characterize the phenotype of *weaver* mice.

Kir3 channel inhibition, induced by intrathecal administration of tertiapin, is pro-convulsant (Mazarati et al., 2006). Moreover, several drugs used in clinics—desimipramine, fluoxetine, haloperidol, thioridazine, pimozide and clozapine—inhibit Kir3 channel activity, and cause seizures as side effect. Conversely, electroconvulsive shock leads to increased expression of Kir3 channels (Pei et al., 1999), which may provide compensatory mechanisms against excessive electrical activity leading to neuroprotection. In support of this hypothesis, stimulation of galanin type 2 receptors that activate GIRK channels prevents kindled epileptogenesis in rats (Mazarati et al., 2006). In conclusion, these studies point out that distinct changes in Kir3 channel activity or availability throughout the brain may result in pro-convulsant or anti-convulsant effects.

## Kir4/Kir5 channelepsy

Kir4.1 subunits (*KCNJ10*; BIR10; Bond et al., 1994; Lagrutta et al., 1996) may form homomeric channels or may polymerize with Kir5.1 (*KCNJ16*) to form heterotetramers (Pessia et al., 1996) highly sensitive to pH (Tucker et al., 2000; Pessia et al., 2001; Casamassima et al., 2003; D'Adamo et al., 2011). Kir4.1 channels are expressed primarily in oligodendrocytes and astrocytes surrounding synapses and blood vessels, mainly in the cortex, thalamus, hippocampus, and brainstem (Takumi et al., 1995; Higashi et al., 2001). Kir4.1 channel activity shows a profound developmental regulation, which correlates with both cell differentiation and the developmental regulation of extracellular K^+^ dynamics (Connors et al., 1982; MacFarlane and Sontheimer, 2000; Neusch et al., 2001). Kir4.1 controls primarily the resting membrane potential of astrocytes, and maintains the extracellular ionic and osmotic environment by promoting K^+^ transport from regions of high [K^+^]_o_, which results from synaptic excitation, to those of low [K^+^]_o_. This polarized transport of K^+^ in astrocytes, referred to as “*spatial buffering of K^+^*” is essential for normal neuronal activity, excitability, and synaptic functions. Among the genes associated with different forms of epilepsy Kir4.1 is receiving increasing interest. Genetic studies have indicated a linkage between missense variations in Kir4.1 and seizure susceptibility (Buono et al., 2004; Connors et al., 2004, Table 1). The DBA/2 mouse strain exhibits a greater susceptibility to induced seizures compared to the C57BL/6 strain. Previous QTL mapping identified the seizure susceptibility locus (*Szs1*) on the distal region of mouse chromosome 1 and further fine mapping studies suggested that a missense variation (T262S) in *Kcnj10* was the likely candidate for this linkage (Ferraro et al., 2004, Table 2). In a second linkage study, a variation in the human *KCNJ10* gene (R271C) was associated with seizure resistance in groups of patients with either focal or generalized epilepsy (Buono et al., 2004). However, a functional study demonstrated that these variations (T262S and R271C) do not produce any observable change in channel function or in predicted channel structure (Shang et al., 2005). It is therefore unlikely that the seizure susceptibility phenotypes associated with these missense variations are caused by changes in the intrinsic functional properties of Kir4.1. However, this study was unable to comprehensively disprove this association, and alterations in Kir4.1 channel activity remain an attractive mechanistic hypothesis. Future investigations willing to prove the association between these variants and seizure susceptibility phenotypes should include examination of how these variants could produce subtle changes in their interaction with cell-specific trafficking or regulatory proteins, or other possible pathways.

Recordings from surgical specimens of patients with intractable epilepsies have demonstrated a reduction of Kir conductance in astrocytes (Bordey and Sontheimer, 1998) and potassium clearance (Jauch et al., 2002). Moreover, *loss-of-function* recessive mutations of *KCNJ10* (Kir4.1) have been recently associated with a disease, named EAST syndrome or SeSAME syndrome, consisting of seizures, ataxia, sensorineural deafness, mental retardation, and renal salt-losing tubulopathy (Bockenhauer et al., 2009; Scholl et al., 2009, Table 1). A number of additional elements substantiate the hypothesis that variants in *KCNJ10* might contribute to brain dysfunction and seizures susceptibility. Conditional knockout mice lacking Kir4.1 exhibit stress-induced seizures, severe ataxia, spongiform vacuolation, axonal swellings, and degeneration, in addition to hearing loss and premature lethality (Neusch et al., 2001; Djukic et al., 2007, Table 2). In Kir4.1 knockout glial cells, no variations in membrane potential were observed during increases in [K^+^]_o_ induced by nerve stimulations (Chever et al., 2010). Hence, it has been proposed that the *loss-of-function* of glial K^+^ conductance would favor extracellular K^+^ accumulation, contributing to neuronal hyperexcitability and epilepsy (Orkand et al., 1966; Chever et al., 2010).

Epilepsy and autism spectrum disorders (ASD) are strongly associated. The prevalence of seizures is highly represented in ASD (5–46%) (Bryson et al., 1988; Hughes and Melyn, 2005), compared with the general population (0.5–1%). The prevalence of autism in the epilepsy population is ~32%, which is about 50 times higher than in the general population (Clarke et al., 2005). An “autism–epilepsy phenotype” has been identified (Tuchman et al., 2009). Recently we reported a mutational screening of *KCNJ10* in 52 children with cryptogenic epilepsy that resulted in the identification of two heterozygous *KCNJ10* mutations in two identical twins (R18Q) and in a 14-year-old child (V84M; Sicca et al., 2011, Table 1). Clinically, the two 8-year-old identical twins showed impaired social interaction, sleep difficulties, hypotonia and both exhibited epileptic spasms within the same 24 h period. Other symptoms, typical of ASD, included clumsiness, absence of speech, severe disorder of social interaction, stereotypies, repetitive behaviors, symptoms of anxiety, depression, obsessive compulsive disorder and intellectual disability (IQ: 58). The 14-year-old child showed normal psychomotor development until 12 months of age, when ASD symptoms such as poor social gaze, no response to name, absence of language development, and withdrawal behaviors became evident. At the age of 6, he experienced complex partial seizures. EEG recordings showed synchronous and asynchronous paroxysmal abnormalities over frontal regions in both hemispheres tending to spread. The functional consequences of these heterozygous mutations were *gain-of-function* of either Kir4.1 or Kir4.1/Kir5.1 channels (Sicca et al., 2011). To date, we have identified several new probands displaying an autism–epilepsy phenotype who carry mutations in *KCNJ10* that cause *gain-of-function* effects, assessed by using astrocytoma cell lines. Collectively, our findings point to a new class of genes that should be examined in autism-epilepsy patients, and disclose novel molecular mechanisms that may increase the susceptibility to this distinct neuropsychiatric phenotype by altering the K^+^ homeostasis in the brain (D'Adamo et al., 2011; Sicca et al., 2011). Indeed, Kir4.1 is the main glial inward conductance, astrocytes make up 90% of all human brain cells, and each astrocyte controls the activity of many thousands of synapses (about 140,000 in the hippocampus; Benarroch, 2009). Co-occurrence of epilepsy and ASD in patients harboring *KCNJ10 gain-of-function* mutations suggests that dysfunction in the astrocytic-dependent K^+^ buffering may be a common mechanism contributing to seizures as well as the core behavioral features of ASD. It has been shown that an isolated episode of local neuronal hyperactivity triggers a large and synchronous Ca^2+^ elevation in closely associated astrocytes. Activated astrocytes signal back to neurons favoring their recruitment into a coherent activity that underlines the hypersynchronous ictal discharge (Gómez-Gonzalo et al., 2010). It is possible that an increased and faster influx of K^+^ into astrocytes through *high functioning* Kir4.1-containing channels may lead, during intense neuronal activity, to larger membrane depolarization and higher intracellular Ca^2+^ elevations in these cells. Ca^2+^ elevations in astrocytes are associated with the release of gliotransmitters, such as glutamate and D-serine, which trigger discharges in neurons, promote local neuronal synchrony and epileptic activity (Parpura et al., 1994; Bezzi et al., 1998; Pasti et al., 2001; Angulo et al., 2004; Fellin et al., 2004; Mothet et al., 2005; Tian et al., 2005). Speculatively, a recurrent neuron-astrocyte-neuron excitatory loop may develop at a restricted brain site, as a consequence of *gain-of-function* of Kir4.1 channels, and contribute to initiation of seizures. These mutations may alter the noradrenergic (NA) system of the brain as well, since Kir4.1/Kir5.1 channels control the excitability of *locus coeruleus* (LC) neurons (D'Adamo et al., 2011b). Indeed, a developmental dysregulation of this LC-NA network (Samuels and Szabadi, 2008) has been suggested to underlie epilepsy. From a therapeutic perspective, these studies indicate that, alike neurons, astrocytes may represent a crucial target for the pharmacological control of abnormal electrical discharge and synaptic function.

## Kir6-K_ATP_-channelepsy

The adenosine triphosphate (ATP)-sensitive K^+^ (K_ATP_) channels are octamers composed of four pore-forming subunits, consisting of Kir6.1 or Kir6.2, with four regulatory sulfonylurea receptors such as SUR1, SUR2A, or SUR2B, which are members of the ATP-binding-cassette transporter family (Aguilar-Bryan et al., 1995; Inagaki et al., 1995; Nichols et al., 1996). The secretion of insulin from pancreatic β-cells is mediated by the closure of these channels caused by increased levels of cytoplasmic ATP. Neuronal K_ATP_ channels are predominantly composed of Kir6.2/SUR1, although Kir6.1/SUR2B and Kir6.2/SUR2B are also found. These channels link the metabolic state of neurons to their excitability by sensing changes in intracellular phosphate potential (i.e., ATP/ADP ratio). DEND syndrome (OMIM 606176) is an inherited disease characterized by **d**evelopmental delay, **e**pilepsy and **n**eonatal **d**iabetes mellitus. Twenty percent of these patients have associated neurologic defects, the most severe of which are generalized epilepsy, marked delay of motor and social development, including late development of speech, and learning difficulties. Numerous *gain-of-function* mutations have been identified in the genes encoding Kir6.2 (*KCNJ11*) or the associated regulatory SUR1 subunit (*ABCC8*) of patients affected by DEND syndrome (Table 1). To date, all mutations in *KCNJ11* that have been characterized functionally, produce marked decrease in the ability of ATP to inhibit the K_ATP_ channel when expressed in heterologous systems or enhance the activatory effects of Mg^2+^-nucleotides. This reduction in ATP sensitivity translates in more fully openings of the channel at physiologically relevant concentrations of ATP, increased K_ATP_ current, hyperpolarization of the β-cell plasma membrane, and consequent suppression of Ca^2+^ influx and insulin secretion (Hattersley and Ashcroft, 2005). Clinical severity of the disorder correlates with the magnitude of shift in the ATP affinity. Sulfonylureas, which block opened channels and restore glucose homeostasis, ameliorate some of the neurological symptoms of DEND syndrome. How K_ATP_ channel over-activity in the central nervous system results in epilepsy is unclear. Whereas insights have been provided on the mechanisms linking loss of K_ATP_ channel function to increased seizure susceptibility. Notably, generalized seizures can be evoked by metabolic stresses such as hypoxia and hypoglycemia. Kir6.2 knockout mice exhibited high-voltage sharp-wave bursts in the EEG recordings, myoclonic jerks followed by severe tonic-clonic convulsion and death upon exposure to hypoxia (Table 2). However, the wild-type mice remained sedated during this challenge and revived normally (Yamada et al., 2001). Substantia nigra pars reticulata (SNr) and its efferents act as a central gating system in the propagation of seizure activity (Iadarola and Gale, 1982). Remarkably, wild-type neurons in brain slices from the substantia nigra pars reticulata (SNr) were hyperpolarized by hypoxia, whereas the membrane potential of Kir6.2 knockout neurons were depolarized by the perfusion with hypoxic solutions. The SNr and its efferents act as a central gating system in the propagation of seizure activity (Iadarola and Gale, 1982). Therefore, hyperpolarization of SNr neurons upon opening of K_ATP_ channels has been proposed to protect against seizure propagation during metabolic stress, although other brain regions could be involved in this process (Yamada et al., 2001).

## Concluding remarks

Solving the puzzle of epilepsy is an extremely difficult task. Here we have put several new pieces in the puzzle by describing novel genetic defects in K^+^ channels and related proteins that underlie distinct human epileptic phenotypes. We have also analyzed critically the new insights in the neurobiology of this disease that have been provided by investigations on valuable animal models of epilepsy. It is becoming increasingly clear that mutations in K^+^ channel genes or perturbations in K^+^ channel function, even in the absence of a primary channel defect, underlie an increased susceptibility to epilepsy. Despite the abundance of genes encoding for K^+^ channels and associated subunits, and their established crucial functional roles, these genes have been greatly overlooked in the search for the causes underlying idiopathic epilepsy. The extremely high diversity of K^+^ channels and the numerous mutations identified in their genes often generate confusion in the classification of the associated diseases. Therefore, we proposed to name the K^+^ channels defects underlying distinct epilepsies as “*K^+^ channelepsies*” and suggested a new classification according to a widely used K^+^ channel nomenclature (Chandy and Gutman, 1993; Kubo et al., 2005; Wei et al., 2005). This original classification could be also adopted to easily unify, identify and describe multiple organ dysfunction related to a single ion channel gene defect (*e.g*., K*x.y*-*phenotype;* Na_v_*x.y*-*phenotype*; Ca_v_*x.y*-*phenotype*) (Catterall et al., 2005a,b). K^+^ channels represent crucial targets for novel pharmacological control of abnormal electrical discharges and synaptic function in the brain. Much greater efforts should thus be made to find new K^+^ channel modulators and gene therapies to ameliorate the symptoms of this devastating disease. On the other hand, the effects of newly developed drugs on the activity of most K^+^ channel types should be tested in order to predict their pro-convulsant side effects. Research on *K^+^ channelepsies* is clearly providing important knowledge on the signaling pathways and circuits involved in epilepsy. The understanding of these “experiments” of *Nature* will also help us to uncover, in a much broader sense, the physiological workings of the human body.

### Conflict of interest statement

The authors declare that the research was conducted in the absence of any commercial or financial relationships that could be construed as a potential conflict of interest.
