# Erratum: K^+^ channelepsy: progress in the neurobiology of potassium channels and epilepsy

**DOI:** 10.3389/fncel.2014.00009

**Published:** 2014-01-30

**Authors:** Maria C. D'Adamo, Luigi Catacuzzeno, Giuseppe Di Giovanni, Fabio Franciolini, Mauro Pessia

**Affiliations:** ^1^Section of Human Physiology, Department of Internal Medicine, Faculty of Medicine, University of PerugiaPerugia, Italy; ^2^Istituto Euro Mediterraneo di Scienza e Tecnologia, IEMESTPalermo, Italy; ^3^Dipartimento di Biologia Cellulare e Ambientale, Università di PerugiaPerugia, Italy; ^4^Department of Physiology and Biochemistry, University of MaltaMsida, Malta

**Keywords:** potassium channels, channelopathies, channelepsy, channelepsies, temporal lobe epilepsy, autism–epilepsy

After our article was published online, we recognized that the list of work being quoted was incomplete. We have included here the following missing reference: Lossin, 2009.
